# Transmission of dominant strains of *Campylobacter jejuni* and *Campylobacter coli* between farms and retail stores in Ecuador: Genetic diversity and antimicrobial resistance

**DOI:** 10.1371/journal.pone.0308030

**Published:** 2024-09-24

**Authors:** Lorena Montero, José L. Medina-Santana, María Ishida, Brian Sauders, Gabriel Trueba, Christian Vinueza-Burgos

**Affiliations:** 1 Instituto de Microbiología, Colegio de Ciencias Biológicas y Ambientales, Universidad San Francisco de Quito, Quito, Ecuador; 2 Unidad de investigación de Enfermedades Transmitidas por Alimentos y Resistencia a los Antimicrobianos (UNIETAR), Facultad de Medicina Veterinaria y Zootecnia, Universidad Central de Ecuador, Quito, Ecuador; 3 Division of Food Laboratory, New York State Department of Agriculture and Markets, Albany, NY, United States of America; University of Tripoli, LIBYA

## Abstract

Thermotolerant *Campylobacter* is an important zoonotic pathogen known for causing gastroenteritis in humans, with poultry as its primary reservoir. A total of 468 samples were collected, of which 335 were chicken carcass samples (representing the food component), and 133 were chicken caeca samples (representing the animal component). These samples underwent culture, with colonies examined under a microscope. Species identification was achieved through multiplex PCR. Additionally, antimicrobial susceptibility profiles were determined using the Kirby-Bauer method, testing for sensitivity to gentamicin, ciprofloxacin, tetracycline, and erythromycin. Additionally, 55 *C*. *jejuni* (62.5%) and 33 *C*. *coli* (37.5%) isolates were selected for whole genome sequencing (WGS). A High prevalence of *Campylobacter* was observed, with rates of 95.5% (n = 127, CI_95%_: 92.5% - 98.5%) in the animal component and 72.5% (n = 243, CI_95%_: 69.9% - 75.1%) in the food component. Specifically, *C*. *jejuni* was detected in 33.1% (n = 42) of poultry farms and 38.3% (n = 93) of chicken carcasses, while *C*. *coli* was found in 64.6% (n = 82) of poultry farms and 60.5% (n = 147) of chicken carcasses. Antimicrobials with the highest rates of resistance (67%-100%) were ciprofloxacin and tetracycline, in both animal and food component isolates. Erythromycin resistance was notable, ranging from 22% to 33%, with only two *C*. *jejuni* isolates from retail were resistant to gentamicin. Furthermore, multidrug resistance was identified in 23% (20 isolates) of the *Campylobacter* isolates. Genetic analysis revealed the presence of fourteen resistance genes in both *C*. *jejuni* and *C*. *coli* isolates, including *tet(O)*, *bla*_OXA-460_, *bla*_OXA-184_, *bla*_OXA-489_, *bla*_OXA-193_, *bla*_OXA-784_, *bla*_OXA-603_, *aph(3’)-IIIa*, *aad9*, *aph(2’’)-If*, *aadE-Cc*, *sat4*, and *ant(6)-Ia*. Additionally, twenty-five plasmids were detected in the 88 *Campylobacter* isolates examined. Interestingly, most isolates also harbored genes encoding putative virulence factors associated with pathogenicity, invasion, adherence, and production of cytolethal distending toxin (cdt): *cheV*, *cheA*, *cheW*, *cheY*, *flaA*, *flgR*, *flaC*, *flaD*, *flgB*, *flgC*, *ciaB*, *ciaC*. The WGS analysis showed the presence of several cgSTs in both animal and food components, with nine of them widely disseminated between components. Moreover, *C*. *coli* and *C*. *jejuni* isolates from different sources presented less than 11 single nucleotide polymorphisms (SNPs), suggesting clonality (16 isolates). Further analysis using SNP tree demonstrated widespread distribution of certain *C*. *jejuni* and *C*. *coli* clones across multiple farms and retail stores. This study presents, for the first-time, insights into the clonality, plasmid diversity, virulence, and antimicrobial resistance (AMR) of thermotolerant *Campylobacter* strains originating from the Ecuadorian poultry industry. The identification of AMR genes associated with the main antibiotics used in the treatment of campylobacteriosis in humans, highlights the importance of the prudent use of antimicrobials in the poultry industry. Additionally, this research remarks the need for regional studies to understand the epidemiology of this pathogen.

## Introduction

Thermotolerant *Campylobacter* belongs to the Campylobacteraceae family and is one of the most common bacterial foodborne pathogens worldwide [1]. The ingestion of as few as 500–800 bacterial cells causes human gastroenteritis [1]. The main *Campylobacter* species associated with campylobacteriosis in humans are *C*. *jejuni* and *C*. *coli* [2]. *C*. *jejuni* is a primary causative agent of foodborne diarrheal disease worldwide [3]. On the other hand, *C*. *coli*, although less prevalent, causes an indistinguishable diarrheal illness [1]. Interestingly, in South America, *Campylobacter coli* has been isolated more frequently, representing about 25% of cases of diarrhea [4].

According to the Centers for Disease Control and Prevention (CDC), thermotolerant *Campylobacter* causes approximately 1.3 million cases of human illness (19.5 per 100,000 inhabitants) in the United States annually [3, 5]. Meanwhile, data from the European Food Safety Authority (EFSA), European States reveals 246,571 cases of campylobacteriosis in 2018. The highest incidence of the disease was associated with the consumption of chicken (37.5%) and turkey meat (28.2%). Consequently, controlling *Campylobacter* in poultry meat has been demonstrated as one of the most effective strategies to diminish the incidence of *Campylobacter* infection in humans [6]. Currently, chicken meat is the first source of protein consumed worldwide with special importance in developing countries where its low cost makes it an affordable option [7]. In fact, in Ecuador, the per capita consumption of chicken meat is 30.14 Kg [8] representing the most consumed type of meat in the country.

In South America, *Campylobacter coli* has been consistently identified in cases of human diarrhea [4, 9, 10], potentially indicating its presence within the food chain [1, 11]. However, few studies report clinical cases [12, 13], prevalence, genetic diversity, and antimicrobial resistance of *Campylobacter* in Ecuador [14–17].

Intestinal campylobacteriosis has an incubation period of 24 to 72 hours [1] and can cause acute bloody or watery diarrhea, fever, weight loss, and cramps [18]. The infection is self-limited most of the time, requiring antibiotic therapy only in severe instances [19]. When treatment is needed, the commonly utilized antimicrobials are macrolide and fluoroquinolone, such as erythromycin and ciprofloxacin, respectively [20]. Additionally, tetracyclines have been recommended as an alternative treatment option [19]. Antimicrobial resistance of *Campylobacter* to first line antibiotics such as ciprofloxacin, has been increasingly reported worldwide [21] prompting the need for constant monitoring of this pathogen [1].

Pulsed-field gel electrophoresis (PFGE) technique, Multilocus sequence typing (MLST), and Restriction fragment length polymorphism (RFLP-flaA) have traditionally used to study the distribution of *Campylobacter* genotypes in various sources and reservoirs [22]. Nevertheless, these methods have limitations that have been overcome by sequence-based protocols (such as MLST and whole genome sequencing-WGS). Although, genetic typing by MLST allows the comparison of results from different laboratories, WGS provides greater genetic resolution. This technique has enabled the study of genes involved in *Campylobacter* motility, adhesion, and invasion into intestinal epithelial cells, as well as genes responsible for the expression of toxins essential for developing of infection in people [23].

In the present study we used a whole genome sequencing approach to understand the genetic diversity, distribution, virulence genes, and AMR profiles of *Campylobacter* isolates originating in poultry farms (animal component) and chicken carcasses at retail (food component).

## Materials and methods

### Study design and sampling

#### Animal component

This study was conducted in the province of Pichincha–Ecuador (0°14’60.00" N -78°34’59.99" W) from November 2017 to September 2018. In total, 133 flocks were investigated. Twenty-five poultry caeca from individual birds were randomly collected from each flock at the slaughterhouse level. These samples were aseptically transported to the laboratory at 4°C. In the laboratory, the caeca were immersed in ethanol for 30 seconds and dried by evaporation. From each cecum, 1g of content was collected in a sterile plastic bag to obtain a pooled sample of 25g [17].

#### Food component

In total, 335 chicken carcasses were collected in traditional street markets, local stores, and supermarkets in Quito city. Each carcass was collected in a sterile bag and transported to the laboratory at 4°C. In the laboratory, 25 g of breast skin from each carcass was collected aseptically for subsequent laboratory analysis. For these samples, no ethical approvals were required under current national regulations. However, the Health Minister of Ecuador reviewed and approved the research protocol in the document MSPCURI 000234–5.

#### Isolation and speciation of *Campylobacter*

The isolation of *Campylobacter* was carried out using an ISO 10272–1:2017 validated culture media [24]. Briefly, 25 g of each sample was homogenized by hand for 1 min. Then, one loop of 10 μL was streaked on a RAPID Campylobacter Medium (BIO-RAD, California, USA) and incubated in microaerobic conditions at 42°C for 48 h using a vacuum chamber filled with a mix of gases (N_2_ 92% and CO_2_ 8%). Presumptive *Campylobacter* colonies presented a brick-red appearance and were observed by microscope after the safranin stain. Two spirally curved colonies were plated on blood agar supplemented with 5% defibrinated sheep blood (BD BBL, Maryland, USA) and incubated under microaerobic conditions at 41°C for 48 h. A subsample of the colonies was used for DNA extraction, and the rest were cryopreserved. DNA was released by the boiling method [25] for PCR identification of *Campylobacter* species [26] (S1 Table in S1 File). Cryopreservation (-80°C) of strains was carried out in sheep blood [17, 27] for later analysis.

#### Antimicrobial susceptibility testing

Antimicrobial susceptibility profiles were determined by the Kirby-Bauer method, in accordance with the European Committee on Antimicrobial Susceptibility testing guidelines (EUCAST) [28]. The evaluated antimicrobials were gentamicin (10μg), ciprofloxacin (5μg), tetracycline (30μg), and erythromycin (15 μg). Interpretation of results was based on the epidemiological cut-off values (ECOFF) recommended by EUCAST [29] (S2 Table in S1 File). The *C*. *jejuni* ATCC 33560 strain was used as a quality control.

#### Whole genome sequencing (WGS) and genome assembly

In order to select a wide diversity of *Campylobacter* genotypes from WGS the following strategy was applied. The selection of isolates from the animal component (poultry farms) was made by skipping a sampling week and considering one isolate per farm. For the food component (chicken carcasses), the first recovered isolate from each retail segment (street markets, local stores, and supermarkets) was selected skipping a sampling week. This selection delivered 88 *Campylobacter* isolates that were WGS (55 *C*. *jejuni* and 33 *C*. *coli*) as described in S3 Table in S1 File.

Extraction of genomic DNA was performed from the 88 selected *Campylobacter* isolates using the Wizard® Genomic DNA Purification kit (Promega, USA) following the manufacturer’s instructions. DNA quantification and quality parameters were also measured using a Quantus fluorometer (Promega, MD) and NanoDrop 2000 UV-Vis (Thermo Fisher Scientific). Whole genome sequencing was performed using the MiSeq platform (Illumina, San Diego, CA) according to FDA GenomeTrakr/CDC Pulse Net protocols in the New York State Department of Agriculture and Markets [30]. Sequence accession numbers are available under BioProject PRJNA788759.

#### Bioinformatics analyses

Reads quality was assessed by FastQC V. 0.11.9 [31], the Adapter/Quality Trimming was performed using BBDuk v.38.84 [32], and assembling of reads was made using SPAdes assembler v.3.15.2 [33]. KmerFinder (www.genomicepidemiology.org) was used to identify genus and species. The tools MLST V. 2.0.9 [34] and cgMLSTFinder v.1.2 [35] from the Center for Genomic Epidemiology (www.genomicepidemiology.org) were used for Multilocus sequence typing (MLST) and Core genome MLST (cgMLST) respectively with default settings.

Pan-genome analysis was conducted for each species using Roary v.3.13.0 [36], where genes identified as core were present in at least 95% of the sequences of the isolates analyzed. Afterward, the SNPs of all core genomes were extracted by SNP-sites v.2.5.1 [37]. Finally, a maximum-likelihood phylogenetic tree with 1,000 bootstrap replicates based on SNP´s was constructed using RaxML-NG v.1.1.0 [38]. The phylogenetic tree was pictured using iTOL v.6 web tool [39]. The number of SNP differences between isolates was quantified using the Snippy program with standard settings [40].

Additionally, AMRFinderPlus v.3.10.24 [41] was used to inquire about mobile genes and point mutations (SNPs) related to Antimicrobial Resistance (AMR). In addition, the ABRicate tool v.1.0 [42] with Virulence Factor Database-VFDB (dated 2022/04/27) were used to identify virulence genes using a threshold of at least 80% for identity and coverage [43].

Plasmid prediction was performed using Platon v.1.6 [44]. Complementary, the identity of the plasmid was accessed using the map to reference tool of Geneious Prime 2022.1.1. (https://www.geneious.com) and NCBI-BLAST (https://blast.ncbi.nlm.nih.gov/Blast.cgi).

### Statistical analysis

Microsoft Excel (2022) was used to calculate the prevalence with 95% confidence intervals.

## Results

### Prevalence

During the study, 468 samples, 133 from feces (animal component) and 335 from carcasses (food component) were analyzed. The prevalence of *Campylobacter* was 95.5% (n = 127, CI_95%_: 92.5% - 98.5%) in the animal component and 72.5% (n = 243, CI_95%_: 69.9% - 75.1%) in the food component. We detected 33.1% (n = 42) and 38.3% (n = 93) of *C*. *jejuni* in poultry farms and chicken carcasses, respectively. On the other hand, *C*. *coli* was found in 64.6% (n = 82) of poultry farms and 60.5% (n = 147) of chicken carcasses. The species of six isolates (three isolates from retail and three from farms) could not be identified by Multiplex PCR (S1 Table in S1 File).

### Antimicrobial resistance

#### Antibiogram

The antimicrobials with the highest resistance rates were ciprofloxacin and tetracycline (67%– 100%). The percentage of resistance was higher in *C*. *jejuni* than in *C*. *coli* from the food and animal components. On the other hand, resistance to erythromycin ranged from 22 to 33%, while only two isolates of *C*. *jejuni* originating from the food component were resistant to gentamicin (Table 1).

**Table 1:** Antimicrobial resistance of *C*. *jejuni* and *C*. *coli* isolates.

Antibiotic	Number (%) of resistant isolates			
	Animal component (poultry farm) n, (%)		Food component (chicken carcasses) n, (%)	
	*C*. *jejuni* (n = 23)	*C*. *coli* (n = 15)	*C*. *jejuni* (n = 32)	*C*. *coli* (n = 18)
**Ciprofloxacin**	23 (100%)	15 (100%)	32 (100%)	17 (94%)
**Tetracycline**	22 (96%)	10 (67%)	30 (94%)	13 (72%)
**Erythromycin**	5 (28%)	5 (33%)	7 (22%)	4 (22%)
**Gentamicin**	0 (0%)	0 (0%)	2 (6%)	0 (0%)

In addition, four resistance profiles in all *C*. *coli* and *C*. *jejuni* isolates were observed. Most of isolates were resistant to ciprofloxacin and tetracycline (63%, n = 55) (S4 Table in S1 File). Multidrug resistance (resistance to more than two classes of antibiotics) was detected in nine (27%) of *C*. *coli* isolates and 11 (20%) of *C*. *jejuni* isolates (S4 Table in S1 File).

### Detection of resistance genes and mutations

Fourteen resistance genes were identified in *C*. *jejuni* and *C*. *coli* isolates including *tet(O)* (resistance to tetracycline), *bla*_OXA-460_, *bla*_OXA-184_, *bla*_OXA-489_, *bla*_OXA-193_, *bla*_OXA-784_, *bla*_OXA-603_ (beta-lactams resistance), and *aph(3’)-IIIa*, *aad9*, *aph(2’’)-If*, *aadE-Cc*, *sat4*, and *ant(6)-Ia* (aminoglycoside resistance) (Table 2).

**Table 2:** Number of *Campylobacter coli* and *C*. *jejuni* isolates with resistance-related mutations and acquired genes.

Antibiotic class	Gene (s)	Point mutation	No. of isolates with detected genes and mutations, n, (%)	
			*C*. *coli* (n = 33)	*C*. *jejuni* (n = 55)
**Fluoroquinolone**	*gyrA*	T86I	32, (97%)	54, (98%)
		T86K	0, (0%)	1, (2%)
**Macrolide**	*rplV*	A103V	0, (0%)	12, (22%)
	*23S*	A2075G	9, (27%)	0, (0%)
**Aminoglycoside**	*rpsL*	K88R	1, (3%)	0, (0%)
		K43R	1, (3%)	0, (0%)
	*aph(2’’)-If*		0, (0%)	1, (2%)
	*aph(3’)-IIIa*		4, (12%)	2, (4%)
	*aadE-Cc*		1, (3%)	0, (0%)
	*ant(6)-Ia*		3, (9%)	0, (0%)
	*sat4*		3, (9%)	0, (0%)
	*aad9*		0, (0%)	1, (2%)
**Betalactams**	*blaOXA-450*		1, (3%)	0, (0%)
	*blaOXA-460*		0, (0%)	8, (15%)
	*blaOXA-489*		4, (12%)	0, (0%)
	*blaOXA-193*		9, (27%)	26, (47%)
	*blaOXA-184*		0, (0%)	5, (9%)
	*blaOXA-603*		0, (0%)	1, (2%)
	*blaOXA-784*		2, (6%)	0, (0%)
**Tetracycline**	*tet(O)*		23, (70%)	52, (95%)

Only two *Campylobacter* (one isolate of *C*. *jejuni* and one isolate of *C*. *coli*) did not present the mutation T86I in *gyrA* gene. In addition, the mutation T86K was identified in only one *C*. *jejuni* isolate. The mutation in the *23S*rRNA at position A2075G linked to the erythromycin resistance was detected in 27.2% (n = 9) of *C*. *coli* isolates. The mutation L22: A103V in the *rplV* gene responsible for erythromycin resistance, was observed in 22% of *C*. *jejuni* isolates (n = 12). Finally, the analysis of the gene encoding the S12 ribosomal protein (*rpsL* gene) related to streptomycin resistance, showed the K88R mutation (one isolate of *C*. *coli*) and the K43R mutation (one isolate of *C*. *coli*) (Table 2).

Most of the *Campylobacter* isolates (except for two *C*. *jejuni* isolates) showed genetic resistance determinants compatible with their resistant phenotype (S5 Table in S1 File). One *C*. *jejuni* isolate that was phenotypically resistant to aminoglycosides carried more than one resistance gene for resistance to this group of antibiotics (*aad9*, *aph(3’)-IIIa*, *aph(2’’)-If*).

### Genomic analysis

**Fig 1:**
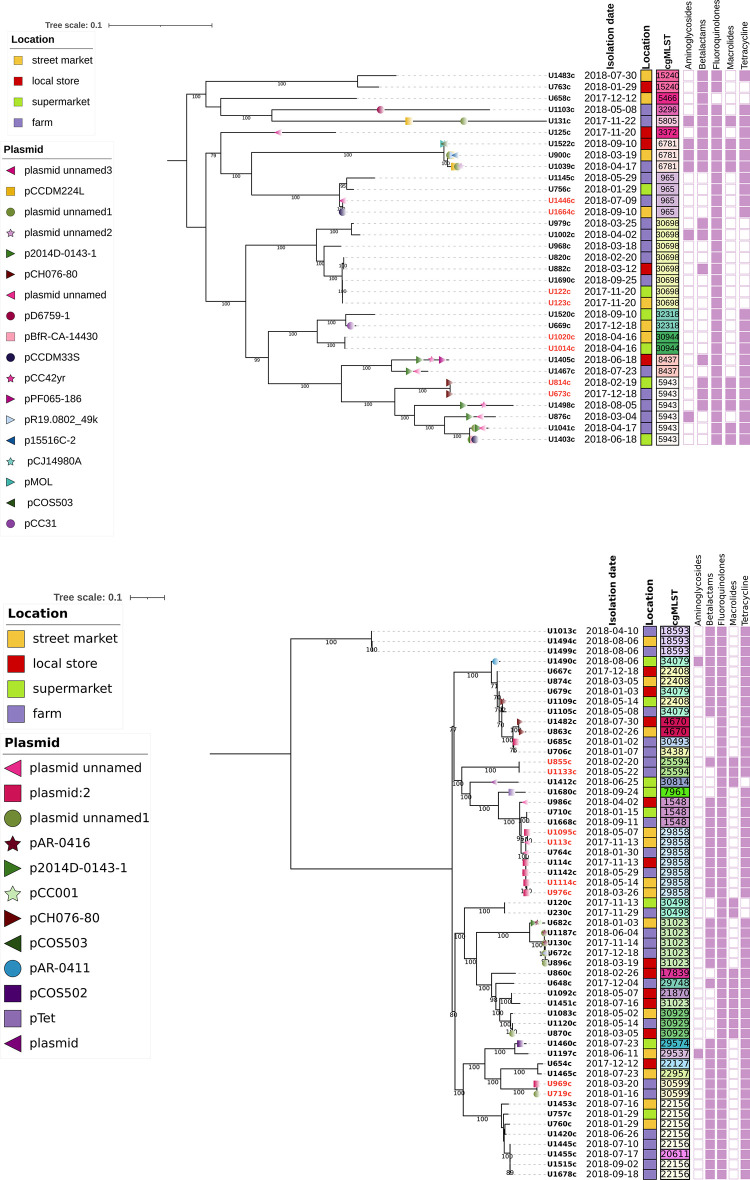
Phylogenetic analysis of *Campylobacter* isolates (a) 33 isolates of *C*. *coli* and (b) 55 isolates of *C*. *jejuni*. The labels show the identification of the isolate. Isolates highlighted in red are isolates with less than 11 SNPs. Isolation date (yyyy/mm/dd). Pink colored blocks represent resistance to antimicrobial classes based on identification of resistance genes. Plasmids are depicted with different figures and colors in each isolate branch.

Core genome sequence type (cgST) designation for the 88 isolates of *Campylobacter* of *C*. *coli* and *C*. *jejuni* is presented in Fig 1A and 1B. The cgMLST analysis showed high genetic diversity among the 55 *C*. *jejuni* and 33 *C*. *coli* strains. However, cgMLST revealed that some *C*. *coli* and *C*. *jejuni* clonal or near clonal isolates (same cgST) were present in different farms and retail stores. We found cgST-22156, cgST-22408, cgST-29858, cgST-30929, cgST-31023, cgST-34079, cgST-965, cgST-6781, cgST-30698 in animal and food components (S6 Table in S1 File). We also found isolates, with less than 11 SNPs of difference in different sources (farms and retail) (S7 Table in S1 File). Furthermore, we found that two *Campylobacter coli* clones that were first isolated in farms (U1446c, U673c) were detected later in retail chicken carcasses (U1664c, U814c) (Fig 1, literal a). Additionally, one clone was found in unrelated farms (Fig 1, literal b, U969c).

MLST analysis using seven housekeeping genes assigned *C*. *jejuni* isolates to 21 known STs and one novel ST. *C*. *coli* isolates were assigned to 11 known STs. (S8 Table in S1 File). The most frequent STs were ST-607 (9 isolates), ST-829 (8 isolates), ST-7669 (7 isolates), and ST-8317 (6 isolates). ST-607, ST-5777, ST-3515, ST-7669, ST-8316, ST-1038, ST-9336, and ST-829 were observed in both components (animal and food). On the other hand, 16STs were observed in isolates originating in either farms (8STs, n = 14) or retail stores (8STs, n = 9) (S8 Table in S1 File).

The 32 isolates of *C*. *coli* in this study belonged to the clonal complex CC-828. One isolate of *C*. *coli* belonged to a not assigned clonal complex (CC) (U658c). Regarding the isolates of *C*. *jejuni*, the most frequent CCs were CC-353 (n = 12), CC-354 (n = 10) and CC-607 (n = 10). Furthermore, four *C*. *jejuni* isolates did not correspond to an assigned CC. Some isolates belonging to the same ST from different components had similar cgSTs assignments (S8 Table in S1 File).

SNP tree analysis also revealed that some *C*. *jejuni* and *C*. *coli* clones were distributed across multiple farms.

### Plasmids identification

A total of 25 plasmids were found, and 47% of isolates (n = 41) carried plasmids (Fig 1A and 1B, S9 Table in S1 File). The pTet plasmid was found in one strain (U1680c) but the tetracycline resistance gene *tetO*, was not present in this plasmid. Furthermore, four *C*. *coli* and one *C*. *jejuni* isolates carried three plasmids, while two *C*. *coli* isolates had four plasmids.

The isolates that carried the plasmids pR19.0802_49k-like (one isolate), pCC31-like (one isolate), pCJ14980A-like (one isolate), pCCDM224L-like (two isolates), and pD6759-1-like (one isolate), harbored the *tetO* gene. Additionally, the *aph(3’)-III* gene related to resistance to aminoglycosides was found in plasmids: pCJ14980A-like (two isolates), pR19.0802_49k-like (one isolate), and pCCDM224L-like (two isolates). Plasmids pCCDM33S-like and pCC42yr-like belonging to type-2 plasmids were found in four isolates of *C*. *coli*.

### Virulence factors

This study detected putative virulence factors associated with pathogenicity, invasion, adherence, and production of the cytolethal distending toxin (*cdt*) *Campylobacter* isolates (S10 Table in S1 File). The putative virulence factors: *cheV*, *cheA*, *cheW*, *cheY*, *flaA*, *flgR*, *flaC*, *flaD*, *flgB*, *flgC*, *ciaB*, *ciaC* were found in most of *C*. *jejuni* and *C*. *coli* isolates (Table 3).

**Table 3:** Number of isolates of *C*. *jejuni* and *C*. *coli*, by putative virulence genes.

Virulence trait	Putative virulence factor	Percentage of positive isolates n, (%)	
		*C*. *coli* (n = 33)	*C*. *jejuni* (n = 55)
**Motility**	*cheA*	28, (85%)	53, (96%)
	*cheV*	28, (85%)	54, (98%)
	*cheW*	28, (85%)	54, (98%)
	*cheY*	28, (85%)	53, (96%)
	*flaA*	21, (64%)	24, (44%)
	*flaB*	18, (55%)	24, (44%)
	*flaC*	28, (85%)	54, (98%)
	*flaD*	28, (85%)	53, (96%)
	*flaG*	30, (91%)	54, (98%)
	*flgA*	2, (6%)	53, (96%)
	*flgB*	28, (85%)	53, (96%)
	*flgC*	29, (88%)	55, (100%)
	*flgR*	32, (97%)	52, (95%)
	*flgS*	32, (97%)	54, (98%)
	*fliA*	28, (85%)	55, (100%)
	*pseD/maf2*	7, (21%)	22, (40%)
	*pseE/maf5*	23, (70%)	47, (85%)
	*pseA*	33, (100%)	54, (98%)
	*ptmA*	23, (70%)	48, (87%)
	*ptmB*	33, (100%)	49, (89%)
	*rpoN*	29, (88%)	54, (98%)
	*maf4*	12, (36%)	26, (47%)
**Adhesion and colonization**	*jlpA*	0, (0%)	53, (96%)
	*porA*	0, (0%)	31, (56%)
	*cadF*	28, (85%)	54, (98%)
	*pebA*	28, 985%)	53, (96%)
**Cytotoxin production**	*cdtA*	0, (0%)	52, (95%)
	*cdtB*	0, (0%)	55, (100%)
	*cdtC*	0, (0%)	55, (100%)
**Invasiveness**	*ciaB*	28, (85%)	53, (96%)
	*ciaC*	28, (85%)	55, (100%)
**Binding and adhesion-LOS**	*wlaN*	0, (0%)	3, (5%)
	*neuA1*	0, (0%)	3, (5%)
	*neuB1*	4, (12%)	3, (5%)
	*neuC1*	4, (12%)	3, (5%)
	*cstIII*	0, (0%)	2, (4%)
**Immune evasion-capsule**	*glf*	0, (0%)	5, (9%)
	*kpsC*	0, (0%)	49, (89%)
	*rfbC*	2, (6%)	26, (47%)
	*kfiD*	0, (0%)	5, (9%)
	*hddA*	11, (33%)	37, (67%)
	*hddC*	0, (0%)	37, (67%)
	*gmhA2*	11, (33%)	37, (67%)
	*fcl*	0, (0%)	7, (13%)

## Discussion

The prevalence of *Campylobacter* in the animal component (poultry farms) was higher (95.5%) compared to the food component (72.5%) (chicken carcasses). However, *Campylobacter* is known to persist in large numbers of fresh foods such as chicken carcasses [45, 46]. Studies conducted in other Latin American countries have reported other rates. For example, the occurrence of *Campylobacter* in carcasses and chicken products was 32.7% in Brazil [47]. Peru also reported a prevalence of 16.7% in carcasses and 26.7% in cecum samples [48]. Besides, the prevalence of *Campylobacter* in poultry-integrated companies ranged from 7% to 10% in Colombia [49]. These variations could be explained by differences in environmental conditions in each country [50, 51], the use of different methodologies for the identification of this pathogen [24], the labile nature of *Campylobacter* in the environment [52], or the high load of *Campylobacter* in feces [53].

In this study, *C*. *coli* was isolated more frequently than *C*. *jejuni* in both components. A higher prevalence of *C*. *coli* (68.7%) over *C*. *jejuni* (18.9%) has been previously reported in Ecuadorian poultry [17]. Other countries in Latin America like Argentina [54], and Peru [55] have also reported a higher prevalence of *C*. *coli*. However, countries like Brazil [56] and Costa Rica [57] have reported a higher prevalence of *C*. *jejuni*. This variation in the ratio of *Campylobacter* species between countries has also been reported in Europe, placing climatic conditions as the probable cause of these observations [58]. Although this statement could be valid in Latin America, more studies are needed to shed light on the regional epidemiology of *C*. *coli* and *C*. *jejuni* [4].

This study revealed resistance rates in *C*. *jejuni* and *C*. *coli* for tetracycline and ciprofloxacin from 30% to 56%. This fact agrees with a previous resistance study where *Campylobacter* was isolated from Ecuadorian broilers at slaughter age [17]. This is also the case in other Latin American countries where similar antibiotic resistance rates have been reported [59, 60]. One of the main factors influencing antimicrobial resistance, especially to fluoroquinolones and tetracyclines, is the use of these antimicrobial agents in animal production. Due to their easy administration and availability without a prescription, these antimicrobials are widely used by farmers without the supervision of a veterinarian [61].

Almost all phenotypes of antimicrobial resistance (AMR) exhibited concordance between phenotypic and genotypic profiles, indicating a strong correlation between genetic determinants. However, in two cases (S5 Table in S1 File) discrepancies were observed. Further analysis suggests that those two cases could be the result of low sequence quality, demonstrating the high levels of AMR predictions reached with the WGS analysis [62].

The most common tetracycline resistance mechanism observed in *Campylobacter* is the protection of the ribosomal binding site. This mechanism is mediated by ribosomal protection proteins encoded by the *tetO* gene, frequently encoded by the plasmid pTet—(type-1) [63]. This gene was identified in 85% of our isolates. However, in this study, only six isolates harboring type-1 plasmid carried the *tetO* gene. Although the presence of *tetO* in the chromosome of *Campylobacter* has been reported previously [64, 65], the presence of this gene in both the chromosome and the plasmids suggests that the gene was present on the chromosome and then transferred to integrated plasmids [63]. These results suggest that processes like transformation, conjugation, and transduction can occur in *Campylobacter* and most likely contribute to the horizontal gene transfer of antibiotic-resistance genes. Previous studies in Ecuador have also reported high frequencies of resistance to ciprofloxacin [14, 17, 66].

Resistance to ciprofloxacin linked to the Thr86-Ile (T86I) substitution in the *gyrA* gene was the most frequent resistance mechanism to quinolones found in this study (n = 86 isolates). Furthermore, this mutation has been commonly observed in fluoroquinolones-resistant *Campylobacter* strains [67, 68]. Other studies conducted in Canada, Senegal, and Brazil have also reported the predominance of this mutation in their ciprofloxacin-resistant chicken isolates [69–71]. On the other hand, the mutation A2075G in 23S rRNA which confers resistance erythromycin [72], was observed in nine *C*. *coli* isolates despite their low phenotypic resistance to macrolides (9%-15%). This mutation has also been reported in Asian countries [73, 74]. Also, the A103V substitution was the major type of substitution in *C*. *jejuni* isolates in this study. This amino acid substitution in the ribosomal proteins L4 and L22 is linked with a low level of macrolide resistance in *Campylobacter* species [72].

The frequency of resistance genes to aminoglycosides in the present study was low (17%) and mainly found in *C*. *coli* isolates: *aph(2’’)-If* (n = 1 isolate), *aph(3’)-IIIa* (n = 6 isolates), *ant(6)-Ia* (n = 3 isolates), *sat4* (n = 3 isolates) and *aad9* (n = 1 isolate). Inversely, a high frequency (73%) of resistance genes of beta-lactamases was found in *C*. *jejuni*, with the *bla*_OXA-193_ gene being the most frequent. The presence of beta-lactamases in a high proportion of *C*. *jejuni* isolates has been documented in some studies [75, 76]. It should be noted that *Campylobacter* exhibits intrinsic resistance to some beta-lactams [77, 78]. However, the genetic determinants of resistance to aminoglycosides and beta-lactamases found in this research should be monitored for possible frequency changes.

The low antimicrobial resistance rates to macrolides and aminoglycosides found in this study suggest that erythromycin and gentamicin can still be used when needed. The detection of AMR genes for the main antibiotics used in the treatment of campylobacteriosis raises concerns and highlights the importance of the prudent use of antimicrobials in Ecuadorian broiler production. In addition, most of the strains in this study showed multidrug resistant profiles (fluoroquinolone, tetracycline, and macrolide) which may reflect the indiscriminate use of these antibiotics [79].

The results of this study underline the importance of poultry in the epidemiology of *Campylobacter* infections as some isolates with the same cgST (cgST5943, cgST965) were found in both animal and food components. It has to be noticed that the mutation rate of thermotolerant *Campylobacter* has been estimated to be 2.07e^-6^ per site per year which is close to 3.5 mutations in the genome per year [80]. Our study identified isolates showing less than 11 SNPs of difference (S7 Table in S1 File), suggesting transmission events among farms of integrated poultry companies and between components (animal and food). Some clonal strains (Fig 1) seemed more successful (capable of thriving in animal intestines, spreading effectively, and surviving in the environments outside the animal host) than others because they were present in different farms and food components at different times (Fig 1A and 1B). It is critical to study whether these successful clones are also causing human disease and it´s severity. The existence of successful *Campylobacter* clones has been described previously [81].

Although having lower molecular resolution than cgMLST, MLST provides valuable information which allows us to compare our data with previous reports. The most frequent *C*. *jejuni* ST was ST-607. Remarkably, this ST has been previously found in chicken isolates in Ecuador [16]. In the same way, other STs (ST-353, ST-462, ST-6091, ST-6244, ST-137, ST-1233, ST-7669, ST-464 and ST-3515) have also been described in *Campylobacter* originated in chickens and other animals in Ecuador [16, 17]. Similarly, seven STs from *C*. *coli* (ST-828, ST-829, ST-902, ST-5777, ST-8316, ST-8317) have been previously found in Ecuador [17]. Some of these STs have been reported in other countries in the broiler production chain (ST-137, ST-3515) and human cases (ST-1233) of gastroenteritis [82–85]. From the 55 *C*. *jejuni* isolates tested, the majority belonged to the CC-353 (n = 12), CC-354 (n = 10) and CC-607 (n = 10), while the 32 out of 33 *C*. *coli* isolates of this study belonged to the CC-828. Predominantly distribution of *C*. *coli* within CC-828 has also been reported in Ecuadorian poultry [17]. Meanwhile, the results in *C*. *jejuni* isolates suggested a high diversity of CCs. This is in accordance with the findings of another local studies [16, 17]. Other less common CCs found in this study (CC-607, CC-574, CC-443) have also been reported in poultry from Korea [86], Thailand [87] and China [88]. Our findings emphasize the importance of studying the epidemiology of *Campylobacter* in low and middle-income countries to learn whether some genotypes might be restricted to a specific source, and whether certain genotypes are most frequently causing human disease.

The pathogenicity of thermotolerant *Campylobacter* is mediated by several virulence factors. The expression of genes that are related to the motility, adhesion, and invasion of *Campylobacter* in intestinal epithelial cells, and toxin production is vital for the colonizing chicken intestines and establishing of infection in humans [23, 89]. The flagellin-coding *flaA* gene, which is the most important for bacterial motility [90] was present in 64% of *C*. *coli* and 44% of *C*. *jejuni* in this study. The low frequency of flagellin genes has already been reported in previous studies [91, 92]. However, it has been reported that the *flaA* gene in *Campylobacter* ranged from 78%-100% [93, 94]. Remarkably, the later studies used PCR techniques that specifically target this gene, while WGS could render gaps in the consensus sequences that could sub-estimate the presence of this genetic determinants [91]. Moreover, one investigation in 40,371 *C*. *jejuni* genomes found that the full length flagellin locus (*flaA* and *flaB*) was present in only 35% of cases [95]. This research proposes that the low identification of these genes by WGS could be explained by recombination events that promote a high variability of these genes within the *C*. *jejuni* genome [95].

The *cadF* gene was detected, in 98% of the *C*. *jejuni* isolates and 85% of *C*. *coli* isolates in this study. The *cadF* gene is responsible for adhesion and influencing microfilament organization in host cells [90]. Similar results were obtained in previous studies in *C*. *jejuni* of poultry origin from Japan, India, and Brazil with the *cadF* gene is present in almost of all isolates [96, 97]. Many virulence factors have been correlated with the invasion of *Campylobacter* into intestinal epithelial cells, including *ciaB* gene (*Campylobacter* invasive antigen B) [90]. This gene was present in or study in 85% of *C*. *coli* and 96% of *C*. *jejuni*. On the other hand, the *cdtA*, *cdtB*, and *cdtC* genes (cytolethal distending toxin-*cdt* operon) are required for the expression of cytotoxins that damage the host’s nuclear DNA and cause cell death [23]. This study found these genes in almost all (99%) *C*. *jejuni* isolates. Comparable findings were previously reported in *Campylobacter* isolates from animals, food, and humans [98]. These findings are in agreement with the notion that the *cdt* operon was more frequently present in *C*. *jejuni* than in *C*. *coli* [99, 100]. It should be noted that genes associated with the occurrence of Guillan Barré syndrome-GBS, including *neuABC*, *wlaN*, and *cstIII*, were detected ranging from 4% to 12% of *Campylobacter* isolates in this study. These genes produce sialyltransferases (molecules resemble mammalian gangliosides), which cause the development of antibodies that could trigger an autoimmune reaction [101]. There are no statistics on Guillain Barré syndrome cases in Ecuador, it would be important to study the association of this syndrome with infections caused by *C*. *jejuni* in the country. The presence of the GBS genes suggests that these isolates could become pathogenic in case of human infection. However, *Campylobacter*´s aptitude to cause human disease is likely multifactorial [90].

Despite the significance of these bacteria as leading causes of foodborne illness, information regarding plasmids in *C*. *jejuni* and *C*. *coli* remains poorly studied in Latin America. Previous investigations focusing on *Campylobacter* isolates from humans, poultry and pigs, revealed plasmid presence in 22% to 64% of isolates [102, 103] which agrees with our results. Moreover, several plasmids identified in this study have yet to be classified within the three recognized classes of *Campylobacter* plasmids [63].

This study showed that *Campylobacter* isolates from poultry caeca and chicken carcasses in Ecuador have high resistance to quinolones and tetracyclines, pathogenicity potential, and diverse genotypes. The coupling of antibiotic resistance and virulence poses a substantial and alarming issue to food safety and public health.

## Conclusion

The present analysis sheds light on the prevalence, antimicrobial resistance patterns, genetic diversity, and virulence factors of *Campylobacter* isolates from poultry caeca and chicken carcasses in Ecuador. The higher prevalence of this pathogen in poultry farms and chicken carcasses underscores the importance of understanding the dynamics of contamination along the food production chain. Moreover, this study reports the predominance of *C*. *coli* over *C*. *jejuni*.

Our findings about the high phenotypic resistance to tetracyclines and quinolones, coupled with the presence of resistance genes and virulence factors, raise concerns regarding food safety and public health. Additionally, the detection of successful clones across farms and food components highlights the potential for transmission of *Campylobacter* to consumers. The analysis of this data emphasizes the importance of prudent antimicrobial use in poultry production and the necessity of an active surveillance of this pathogen in Ecuador.

## Supporting information

S1 File This file contains supporting tables. (DOCX)

S2 File Breakpoints. (PDF)

S3 File Supporting information for each isolate. (PDF)
