# Supplementary material for: Transmission of dominant strains of Campylobacter jejuni and Campylobacter coli between farms and retail stores in Ecuador: Genetic diversity and antimicrobial resistance
Source: PLoS One. 2024 Sep 24;19(9):e0308030. doi: 10.1371/journal.pone.0308030 (PMC11421796; doi:10.1371/journal.pone.0308030)
Supplement: S1 File — (DOCX) [file pone.0308030.s001.docx]

**Supporting Information**

**Table S1.** Number of isolates identified by Multiplex PCR

| **Component/Specie** | **Food component (chicken carcasses)** | **Animal component (poultry farm)** |
| --- | --- | --- |
| *C. coli* | 147 | 82 |
| *C. jejuni* | 93 | 42 |
| Negative | 92 | 6 |
| Specie is not identified | 3 | 3 |
| **Total** | 335 | 133 |

**Table S2.** Breakpoints (epidemiological cut-off values ECOFF) used for determination of the antimicrobial resistance of *C. jejuni* and *C. coli*.

| **Antibiotic (disk concentration)** | **Zone diameter**  **(mm)** | |
| --- | --- | --- |
|  | **S≥** | **R<** |
| Gentamicin (10µg), *C. jejuni* | 20 | 20 |
| Ciprofloxacin (5µg), *C. coli*  and *C. jejuni* | 26 | 26 |
| Tetracycline (30µg), *C. coli*  and *C. jejuni* | 30 | 30 |
| Erythromycin (15µg),  *C. jejuni* | 22 | 22 |
| Erythromycin (15µg). *C. coli* | 24 | 24 |

**Table S3.** Number of selected isolates for the study according to their specie and component

| **Specie** | **Animal component (poultry farm)** | **Food component (chicken carcasses)** | **Total** |
| --- | --- | --- | --- |
| ***C. coli*** | 15 | 18 | 33 |
| ***C. jejuni*** | 23 | 32 | 55 |

**Table S4**. Antimicrobial resistance profile of *C. jejuni* and *C. coli* isolates

| **Resistance profiles** | **Number of isolates (%)** | |
| --- | --- | --- |
|  | ***C. coli* (n=33)** | ***C. jejuni* (n=55)** |
| CIP-TE | 14 (42%) | 41 (75%) |
| CIP-ERY | 0 (0%) | 3 (5%) |
| CIP-TE-ERY^a^ | 9 (27%) | 9 (16%) |
| CIP-TE-GM^a^ | 0 (0%) | 2 (4%) |

GM, Gentamicin; CIP, Ciprofloxacin; TE, Tetracycline; ERY Erythromycin

a.Multidrug resistance isolates

**Table S5.** Comparison of phenotypic AMR with resistance determinant

| **Antibiotic family** | **Phenotypic resistance**  **Couts (%)** | | **Genetic determinant of resistance**  **Couts (%)** | | **Phenotypic and genotypic resistance concordance (%)** | |
| --- | --- | --- | --- | --- | --- | --- |
|  | *C. coli* (n=33) | *C. jejuni* (n=55) | *C. coli* (n=33) | *C. jejuni* (n=55) | *C. coli* | *C. jejuni* |
| **Fluoroquinolones** | 32, (97) | 55, (100) | 32, (97) | 54, (98) | 100 | 98 |
| **Tetracycline** | 23, (70) | 52, (95) | 23, (70) | 51, (93) | 100 | 98 |
| **Macrolides** | 9, (27) | 12, (22) | 9, (27) | 12, (22) | 100 | 100 |
| **Aminoglycosides** | 0 | 2, (4) | 0 | 2, (4) | NA | 100 |

NA= not applicable

**Table S6**. Number of isolates of *C.jejuni* and *C.coli* by cgST in animal (poultry farm) and food component ( chicken carcasses)

| **Specie** | **cgST** | **Animal component n, (%)** | **Food component n, (%)** | **Total n, (%)** |
| --- | --- | --- | --- | --- |
| ***C.coli* (n=33)** | 965 | 2, (6%) | 2, (6%) | 4, (12%) |
|  | 3296 | 1, (3%) | 0, (0%) | 1, (3%) |
|  | 3372 | 0, (0%) | 1, (3%) | 1, (3%) |
|  | 5466 | 0, (0%) | 1, (3%) | 1, (3%) |
|  | 5805 | 1, (3%) | 0, (0%) | 1, (3%) |
|  | 5943 | 4, (12%) | 2, (6%) | 6, (18%) |
|  | 6781 | 1, (3%) | 2, (6%) | 3, (9%) |
|  | 8437 | 1, (3%) | 1, (3%) | 2, (6%) |
|  | 15240 | 0, (0%) | 2, (6%) | 2, (6%) |
|  | 30698 | 5, (15%) | 3, (9%) | 8, (24%) |
|  | 30944 | 0, (0%) | 2, (6%) | 2, (6%) |
|  | 32318 | 0, (0%) | 2, (6%) | 2, (6%) |
| ***C.jejuni* (n=55)** | 1548 | 1, (2%) | 2, (4%) | 3, (5%) |
|  | 4670 | 0, (0%) | 2, (4%) | 2, (4%) |
|  | 7961 | 0, (0%) | 1, (2%) | 1, (2%) |
|  | 17839 | 0, (0%) | 1, (2%) | 1, (2%) |
|  | 18593 | 2, (4%) | 1, (2%) | 3, (5%) |
|  | 20611 | 1, (2%) | 0, (0%) | 1, (2%) |
|  | 21870 | 0, (0%) | 1, (2%) | 1, (2%) |
|  | 22127 | 0, (0%) | 1, (2%) | 1, (2%) |
|  | 22156 | 4, (7%) | 3, (5%) | 7, (13%) |
|  | 22408 | 0, (0%) | 3, (5%) | 3, (5%) |
|  | 22957 | 0, (0%) | 1, (2%) | 1, (2%) |
|  | 25594 | 2, (4%) | 0, (0%) | 2, (4%) |
|  | 29537 | 0, (0%) | 1, (2%) | 1, (2%) |
|  | 29574 | 0, (0%) | 1, (2%) | 1, (2%) |
|  | 29748 | 1, (2%) | 0, (0%) | 1, (2%) |
|  | 29858 | 2, (4%) | 5, (9%) | 7, (13%) |
|  | 30493 | 1, (2%) | 0, (0%) | 1, (2%) |
|  | 30498 | 1, (2%) | 1, (2%) | 2, (4%) |
|  | 30599 | 2, (4%) | 0, (0%) | 2, (4%) |
|  | 30814 | 0, (0%) | 1, (2%) | 1, (2%) |
|  | 30929 | 1, (2%) | 2, (4%) | 3, (5%) |
|  | 31023 | 3, (5%) | 3, (5%) | 6, (11%) |
|  | 34079 | 1, (2%) | 2, (4%) | 3, (5%) |
|  | 34387 | 1, (2%) | 0, (0%) | 1, (2%) |

**Table S7.** Number of isolates from different components with less than 11 SNPs

| **Specie** | **Isolates** | **cgST** | **SNP difference** | **Source** |
| --- | --- | --- | --- | --- |
| ***C.coli*** | U122c | 30698 | 9 | food component |
|  | U123c | 30698 |  | food component |
|  | U1020c | 30944 | no SNP | food component |
|  | U1014c | 30944 |  | food component |
|  | U814c | 5943 | 2 | food component |
|  | U673c | 5943 |  | animal component |
|  | U1446c | 965 | 7 | animal component |
|  | U1664c | 965 |  | food component |
| ***C.jejuni*** | U719c | 30599 | 4 | animal component |
|  | U969c | 30599 |  | animal component |
|  | U1133c | 25594 | 11 | animal component |
|  | U855c | 25594 |  | animal component |
|  | U113c | 29858 | 3 | food component |
|  | U1095c | 29858 |  | food component |
|  | U976c | 29858 | 7 | food component |
|  | U1114c | 29858 |  | food component |

**Table S8**. Number of isolates of *C. jejuni* and *C. coli*, by sequence type and clonal complex from animal and food component

| **ST/CC** | ***C. coli* (n=33)** | | ***C. jejuni* (n=55)** | |
| --- | --- | --- | --- | --- |
|  | **Animal component** | **Food component** | **Animal component** | **Food component** |
| **ST-51** |  |  | **2** |  |
| CC-443 |  |  | 2 |  |
| **ST-137** |  |  | **2** | **1** |
| CC-45 |  |  | 2 | 1 |
| **ST-353** |  |  | **1** |  |
| CC-353 |  |  | 1 |  |
| **ST-462** |  |  |  | **1** |
| CC-353 |  |  |  | 1 |
| **ST-464** |  |  | **1** |  |
| CC-464 |  |  | 1 |  |
| **ST-607** |  |  | **2** | **7** |
| CC-607 |  |  | 2 | 7 |
| **ST-825** |  | **2** |  |  |
| CC-828 |  | 2 |  |  |
| **ST-828** | **2** |  |  |  |
| CC-828 | 2 |  |  |  |
| **ST-829** | **3** | **5** |  |  |
| ST-828 | 3 | 5 |  |  |
| **ST-902** |  | **2** |  |  |
| CC-828 |  | 2 |  |  |
| **ST-1036** |  |  |  | **1** |
| CC-353 |  |  |  | 1 |
| **ST-1038** |  |  | **1** | **1** |
| CC-354 |  |  | 1 | 1 |
| **ST-1055** | **1** | **2** |  |  |
| CC-828 | 1 | 2 |  |  |
| **ST-1107** | **1** |  |  |  |
| CC-828 | 1 |  |  |  |
| **ST-1233** |  |  |  | **2** |
| CC-353 |  |  |  | 2 |
| **ST-1359** |  |  | **2** |  |
| CC-21 |  |  | 2 |  |
| **ST-1581** |  | **1** |  |  |
| not reported |  | 1 |  |  |
| **ST-3515** |  |  | **1** | **2** |
| CC-353 |  |  | 1 | 2 |
| **ST-5401** |  |  |  | **1** |
| CC-354 |  |  |  | 1 |
| **ST-5777** | **2** | **2** |  |  |
| CC-828 | 2 | 2 |  |  |
| **ST-6091** |  |  |  | **2** |
| not reported |  |  |  | 2 |
| **ST-6244** |  |  | **4** |  |
| CC-574 |  |  | 4 |  |
| **ST-7356** |  |  |  | **1** |
| not reported |  |  |  | 1 |
| **ST-7669** |  |  | **2** | **5** |
| CC-354 |  |  | 2 | 5 |
| **ST-8316** | **1** | **2** |  |  |
| CC-45 | 1 |  |  |  |
| CC-828 |  | 2 |  |  |
| **ST-8317** | **4** | **2** |  |  |
| CC-828 | 4 | 2 |  |  |
| **ST-9336** |  |  | **1** | **3** |
| CC-353 |  |  | 1 | 3 |
| **ST-10237** |  |  |  | **1** |
| CC-354 |  |  |  | 1 |
| **ST-10241** |  |  |  | **1** |
| not reported |  |  |  | 1 |
| **ST-10413** |  |  |  | **1** |
| CC-574 |  |  |  | 1 |
| **ST-10588** | **1** |  |  |  |
| CC-828 | 1 |  |  |  |
| **ST-10618** |  |  | **1** |  |
| CC-607 |  |  | 1 |  |
| **ST-new 1** |  |  | **3** | **1** |
| CC new |  |  | 3 | 1 |

*STnew allelic profile: *aspA* (8), *glnA* (113), *gltA* (5), *glyA* (121), *pgm* (606), *tkt* (25), *uncA* (6)

**Table S9**. Number of *C. jejuni* and *C. coli* isolates per plasmid

| **Plasmid** | ***C. coli* (n=33)** | ***C. jejuni* (n=55)** |
| --- | --- | --- |
| **pCCDM224L-like** | 4 | 0 |
| **plasmid unnamed1-like** | 5 | 6 |
| **plasmid unnamed2-like** | 1 | 0 |
| **p2014D-0143-1-like** | 8 | 1 |
| **plasmid unnamed-like** | 3 | 8 |
| **plasmid:2-like** | 0 | 7 |
| **pD6759-1-like** | 1 | 0 |
| **pAR-0416-like** | 0 | 4 |
| **pCH076-80-like** | 2 | 3 |
| **plasmid unnamed3-like** | 2 | 0 |
| **pBfR-CA-14430-like** | 1 | 0 |
| **pCCDM33S-like** | 2 | 0 |
| **pCC42yr-like** | 2 | 0 |
| **pPF065-186-like** | 1 | 0 |
| **Plasmid-like** | 0 | 1 |
| **pCOS502-like** | 0 | 1 |
| **pAR-0411-like** | 0 | 1 |
| **pCJ14980A-like** | 1 | 0 |
| **pMOL-like** | 1 | 0 |
| **pCOS503-like** | 1 | 1 |
| **pTet-like** | 0 | 1 |
| **pCC31-like** | 1 | 0 |
| **pCC001-like** | 0 | 1 |
| **pR19.0802_49k-like** | 1 | 0 |
| **p15516C-2-like** | 1 | 0 |

**Table S10**. Virulence genes found in *C. coli* and *C. jejuni* from animal and food component isolates

| **Virulence trait** | **Putative virulence factors** | **Sampling location** | | |
| --- | --- | --- | --- | --- |
|  |  | **Animal component n (%)** | **Food component n (%)** | **Total (n=88)** |
| **Motility** | *Maf4* | 13, (34%) | 25, (66%) | 38 |
|  | *cheA* | 36, (44%) | 45, (56%) | 81 |
|  | *cheV* | 36, (44%) | 46, (56%) | 82 |
|  | *cheW* | 36, (44%) | 46, (56%) | 82 |
|  | *cheY* | 36, (44%) | 27, (33%) | 81 |
|  | *flaA* | 21, (47%) | 24, (53%) | 45 |
|  | *flaB* | 22, (52%) | 20, (48%) | 42 |
|  | *flaC* | 36, (44%) | 46, (56%) | 82 |
|  | *flaD* | 36, (44%) | 45, (56%) | 81 |
|  | *flaG* | 37, (44%) | 47, (56%) | 84 |
|  | *flgA* | 23, (42%) | 32, (58%) | 55 |
|  | *flgB* | 36, (44%) | 45, (56%) | 81 |
|  | *flgC* | 36, (43%) | 48, (57%) | 84 |
|  | *flgR* | 38, (45%) | 46, (55%) | 84 |
|  | *flgS* | 38, (44%) | 48, (56%) | 86 |
|  | *fliA* | 36, (43%) | 47, (57%) | 83 |
|  | *pseD/maf2* | 12, (41%) | 17, (59%) | 29 |
|  | *pseE/maf5* | 32, (46%) | 38, (54%) | 70 |
|  | *ptmA* | 33, (46%) | 38, (54%) | 71 |
|  | *ptmB* | 36, (44%) | 46, (56%0 | 82 |
|  | *rpoN* | 37, (45%) | 46, (55%) | 83 |
|  | *pseA* | 38, (44%) | 49, (56%) | 87 |
| **Adhesion and colonization** | *cadF* | 36, (44%) | 46, (56%) | 82 |
|  | *jlpA* | 23, (43%) | 30, (57%) | 53 |
|  | *porA* | 15, (48%) | 16, (52%) | 31 |
|  | *pebA* | 36, (44%) | 45, (56%) | 81 |
| **Cytotoxin production** | *cdtA* | 21, (40%) | 31, (60%) | 52 |
|  | *cdtB* | 23, (42%) | 32, (58%) | 55 |
|  | *cdtC* | 23, (42%) | 32, (58%) | 55 |
| **Invasiveness** | *ciaB* | 36, (44%) | 45, (56%) | 81 |
|  | *ciaC* | 36, (43%) | 47, (57%) | 83 |
| **Binding and adhesion-LOS** | *cstIII* | 0, (0%) | 2, (100%) | 2 |
|  | *neuA1* | 0, (0%) | 3, (100%) | 3 |
|  | *neuB1* | 2, (29%) | 5, (71%) | 7 |
|  | *neuC1* | 2, (29%) | 5, (71%) | 7 |
|  | *wlaN* | 0, (0%) | 3, (100%) | 3 |
| **Immune evasion-capsule** | *fcl-* | 3, (43%) | 4, (57%) | 7 |
|  | *glf* | 3, (60%) | 2, (40%) | 5 |
|  | *gmhA2* | 20, (42%) | 28, (58%) | 48 |
|  | *hddA* | 20, (42%) | 28, (58%) | 48 |
|  | *hddC* | 14, (38%) | 23, (62%) | 37 |
|  | *kfiD* | 3, (60%) | 2, (40%) | 5 |
|  | *kpsC* | 21, (43%) | 28, (57%) | 49 |
|  | *rfbC* | 11, (39%) | 17, (61%) | 28 |
