# Supplementary material for: Transmission of dominant strains of Campylobacter jejuni and Campylobacter coli between farms and retail stores in Ecuador: Genetic diversity and antimicrobial resistance
Source: PLoS One. 2024 Sep 24;19(9):e0308030. doi: 10.1371/journal.pone.0308030 (PMC11421796; doi:10.1371/journal.pone.0308030)
Supplement: S2 File — (PDF) [file pone.0308030.s002.pdf]

Supplementary file 2

Breakpoints (epidemiological cut-off values ECOFF) used for determination of the antimicrobial resistance of *C. jejuni* and *C. coli*.

**Antibiotic (disk concentration)**	**Zone diameter (mm)**	
	**S≥**	**R<**
Gentamicin (10µg), *C. jejuni*	20	20
Ciprofloxacin (5µg), *C. coli* and *C. jejuni*	26	26
Tetracycline (30µg), *C. coli* and *C. jejuni*	30	30
Erythromycin (15µg), *C. jejuni*	22	22
Erythromycin (15µg). *C. coli*	24	24
