# Supplementary material for: Transmission of dominant strains of Campylobacter jejuni and Campylobacter coli between farms and retail stores in Ecuador: Genetic diversity and antimicrobial resistance
Source: PLoS One. 2024 Sep 24;19(9):e0308030. doi: 10.1371/journal.pone.0308030 (PMC11421796; doi:10.1371/journal.pone.0308030)
Supplement: S3 File — (PDF) [file pone.0308030.s003.pdf]

Number	Isolate	Specie	location	plasmid	Biosample	ST	CC	cgST
1	U113c	*C. jejuni*	street market	plasmid unnamed	SAMN25161121	7669	ST-354	29858
2	U114c	*C. jejuni*	local store	plasmid unnamed, plasmid:2	SAMN25248594	7669	ST-354	29858
3	U120c	*C. jejuni*	supermarket		SAMN25247703	464	ST-464	30498
4	U122c	*C. coli*	supermarket		SAMN25337492	829	ST-828	30698
5	U123c	*C. coli*	street market		SAMN25337459	829	ST-828	30698
6	U125c	*C. coli*	local store	plasmid unnamed3	SAMN25337457	1055	ST-828	3372
7	U130c	*C. jejuni*	farm	plasmid unnamed1, pAR-0416	SAMN25248607	new	new	31023
8	U131c	*C. coli*	farm	pCCDM224L, plasmid unnamed1	SAMN25337456	1107	ST-828	5805
9	U230c	*C. jejuni*	farm		SAMN25706220	464	ST-464	30498
10	U654c	*C. jejuni*	local store		SAMN25248605	6091	NR	22127
11	U658c	*C. coli*	street market		SAMN25247708	1581	NR	5466
12	U679c	*C. jejuni*	local store		SAMN25248583	607	ST-607	34079
13	U682c	*C. jejuni*	street market	p2014D-0143-1, pAR-0416, pCC001	SAMN25248586	7356	NR	31023
14	U706c	*C. jejuni*	farm		SAMN25247691	607	ST-607	34387
15	U710c	*C. jejuni*	supermarket		SAMN25248603	1038	ST-354	1548
16	U719c	*C. jejuni*	farm	plasmid unnamed1	SAMN25247702	1359	ST-21	30599
17	U757c	*C. jejuni*	supermarket		SAMN25247699	9336	ST-353	22156
18	U760c	*C. jejuni*	street market		SAMN25248587	9336	ST-353	22156
19	U764c	*C. jejuni*	farm	plasmid unnamed	SAMN25247701	7669	ST-354	29858
20	U855c	*C. jejuni*	farm		SAMN25247710	51	ST-443	25594
21	U860c	*C. jejuni*	local store			462	ST-353	17839
22	U863c	*C. jejuni*	street market	pCH076-80	SAMN25248588	607	ST-607	4670
23	U870c	*C. jejuni*	local store	plasmid unnamed1	SAMN25705791	3515	ST-353	30929
24	U874c	*C. jejuni*	street market		SAMN25248599	607	ST-607	22408
25	U896c	*C. jejuni*	local store	plasmid unnamed1	SAMN25248589	new	new	31023
26	U969c	*C. jejuni*	farm	plasmid:2	SAMN25247697	1359	ST-21	30599
27	U1013c	*C. jejuni*	farm		SAMN23824099	137	ST-45	18593

28	U1039c	*C. coli*	farm	pCCDM224L, plasmid unnamed1, plasmid unnamed2	SAMN25248611	8316	ST-828	6781
29	U1083c	*C. jejuni*	street market		SAMN25248609	3515	ST-353	30929
30	U1109c	*C. jejuni*	supermarket	pAR-0416, pCH076-80		607	ST-607	22408
31	U1105c	*C. jejuni*	farm		SAMN25248597	607	ST-607	34079
32	U1114c	*C. jejuni*	street market	plasmid unnamed, plasmid:2	SAMN25248604	7669	ST-354	29858
33	U1187c	*C. jejuni*	farm	plasmid unnamed1, pAR-0416	SAMN25248591	new	new	31023
34	U1197c	*C. jejuni*	street market		SAMN25247690	1036	ST-353	29537
35	U1420c	*C. jejuni*	farm		SAMN25248606	9336	ST-353	22156
36	U1453c	*C. jejuni*	street market		SAMN25248585	9336	ST-353	22156
37	U1455c	*C. jejuni*	farm		SAMN25247711	6244	ST-574	20611
38	U1494c	*C. jejuni*	street market		SAMN25248598	137	ST-45	18593
39	U1499c	*C. jejuni*	farm		SAMN25248601	137	ST-45	18593
40	U1668c	*C. jejuni*	farm			1038	ST-354	1548
41	U648c	*C. jejuni*	farm		SAMN25247695	353	ST-353	29748
42	U667c	*C. jejuni*	local store		SAMN25247704	607	ST-607	22408
43	U672c	*C. jejuni*	farm	plasmid unnamed1, pCOS503	SAMN25248593	new	new	31023
44	U685c	*C. jejuni*	farm	plasmid:2	SAMN25247712	10618	ST-607	30493
45	U673c	*C. coli*	farm	p2014D-0143-1, pCH076-80	SAMN25337454	8317	ST-828	5943
46	U756c	*C. coli*	supermarket		SAMN25337461	5777	ST-828	965
47	U763c	*C. coli*	local store		SAMN25337449	902	ST-828	15240
48	U820c	*C. coli*	farm		SAMN25337458	829	ST-828	30698
49	U979c	*C. coli*	farm		SAMN25248602	828	ST-828	30698
50	U976c	*C. jejuni*	street market	plasmid unnamed, plasmid:2	SAMN25247705	7669	ST-354	29858
51	U876c	*C. coli*	farm	p2014D-0143-1, plasmid unnamed	SAMN25337477	8317	ST-828	5943
52	U968c	*C. coli*	farm		SAMN25337448	829	ST-828	30698

53	U1014c	*C. coli*	supermarket			825	ST-828	30944
54	U1002c	*C. coli*	farm		SAMN24271454	828	ST-828	30698
55	U1041c	*C. coli*	farm	plasmid unnamed1, p2014D-0143-1, plasmid unnamed	SAMN25337450	8317	ST-828	5943
56	U1120c	*C. jejuni*	farm		SAMN25247713	3515	ST-353	30929
57	U1133c	*C. jejuni*	farm		SAMN25247709	51	ST-443	25594
58	U1142c	*C. jejuni*	farm	plasmid unnamed, plasmid:2	SAMN25247696	7669	ST-354	29858
59	U1092c	*C. jejuni*	local store			1233	ST-353	21870
60	U1095c	*C. jejuni*	street market	plasmid unnamed, plasmid:2	SAMN25247706	7669	ST-354	29858
61	U1103c	*C. coli*	farm	pD6759-1	SAMN25337462	10588	ST-828	3296
62	U1145c	*C. coli*	farm		SAMN25248600	5777	ST-828	965
63	U1412c	*C. jejuni*	supermarket	plasmid	SAMN25248584	10241	NR	30814
64	U1403c	*C. coli*	supermarket	plasmid unnamed1, p2014D-0143-1, pBfR-CA-14430, pCCDM33S	SAMN25337479	8317	ST-828	5943
65	U1405c	*C. coli*	local store	p2014D-0143-1, pCC42yr, pPF065-186	SAMN25337490	1055	ST-828	8437
66	U1490c	*C. jejuni*	supermarket	pAR-0411	SAMN25247700	607	ST-607	34079
67	U1445c	*C. jejuni*	farm		SAMN25706221	6244	ST-574	22156
68	U1460c	*C. jejuni*	supermarket	pCOS502	SAMN25248596	10413	ST-574	29574
69	U1465c	*C. jejuni*	street market		SAMN25247698	6091	NR	22957
70	U1482c	*C. jejuni*	local store	pCH076-80	SAMN25248595	607	ST-607	4670
71	U1446c	*C. coli*	farm	plasmid unnamed3	SAMN25337491	5777	ST-828	965
72	U1467c	*C. coli*	farm	p2014D-0143-1, plasmid unnamed	SAMN25337465	1055	ST-828	8437
73	U1515c	*C. jejuni*	farm		SAMN25248592	6244	ST-574	22156
74	U1678c	*C. jejuni*	farm		SAMN25247694	6244	ST-574	22156
75	U1520c	*C. coli*	supermarket		SAMN25337452	829	ST-828	32318
76	U1680c	*C. jejuni*	supermarket	pTet	SAMN25248590	10237	ST-574	7961

77	U900c	*C. coli*	street market	pCCDM224L, plasmid unnamed1, pR19.0802_49k, p15516C-2		8316	ST-828	6781
78	U986c	*C. jejuni*	local store	plasmid unnamed		1038	ST-354	1548
79	U1451c	*C. jejuni*	local store			1233	ST-353	31023
80	U1498c	*C. coli*	farm	p2014D-0143-1, pCC42yr	SAMN25337464	8317	ST-828	5943
81	U1522c	*C. coli*	local store	pCJ14980A, pMOL, pCOS503	SAMN25337463	8316	ST-828	6781
82	U1664c	*C. coli*	street market	pCCDM33S	SAMN25337455	5777	ST-828	965
83	U1690c	*C. coli*	farm		SAMN25337478	829	ST-828	30698
84	U1483c	*C. coli*	street market		SAMN25248608	902	ST-828	15240
85	U669c	*C. coli*	street market	pCC31	SAMN25247714	829	ST-828	32318
86	U814c	*C. coli*	supermarket	p2014D-0143-1, pCH076-80		8317	ST-828	5943
87	U882c	*C. coli*	local store		SAMN25247707	829	ST-828	30698
88	U1020c	*C. coli*	street market		SAMN25248610	825	ST-828	30944
